# Enhanced Biocontrol of Cotton Verticillium Wilt Through Optimized Solid-State Fermentation of *Myxococcus fulvus* KS01 Using Insect Frass as a Matrix

**DOI:** 10.3390/microorganisms14030610

**Published:** 2026-03-09

**Authors:** Jian Han, Yongcheng Chen, Qiang Sheng, Wei Lu, Ming Luo, Benzhong Fu, Deying Ma

**Affiliations:** 1 Department of Plant Pathology, College of Agronomy, Xinjiang Agricultural University, Urumqi 830052, China; 18167821398@163.com (Y.C.); teerakon@sina.com (W.L.); luomingxjau@sina.com (M.L.); benzhongf@yahoo.com (B.F.); 2 Key Laboratory of Prevention and Control of Invasive Alien Species in Agriculture & Forestry of the North-Western Desert Oasis (Co-Construction by Ministry and Province), Ministry of Agriculture and Rural Affairs, Urumqi 830052, China; 3 Key Laboratory of the Pest Monitoring and Safety Control of Crops and Forests of the Xinjiang Uygur Autonomous Region, Urumqi 830052, China; 4 Academy of Agricultural Sciences, Xinjiang Bayingolin Mongol Autonomous Prefecture, Korla 841000, China; xjssqq@sina.com

**Keywords:** *Myxococcus fulvus* KS01, cotton Verticillium wilt, fermentation optimization, response surface methodology, biological control

## Abstract

Cotton Verticillium wilt, caused by *Verticillium dahliae*, is a devastating soil-borne disease that severely limits global cotton production. While *Myxococcus fulvus* KS01 has demonstrated potent antagonistic activity and multi-functional biocontrol effects against *V. dahliae*, its practical application has been hindered by low myxospore yields and inconsistent efficacy in initial solid-state fermentation (SSF). This study aimed to optimize the SSF process for strain KS01 to maximize myxospore production and systematically evaluate its biocontrol efficacy against Verticillium wilt. Using a mixture of wheat straw and *Protaetia brevitarsis* frass (an agricultural byproduct) as the base substrate, we utilized single factor experiments and Response Surface Methodology (RSM) to optimize nutritional supplements and fermentation parameters. The optimized SSF process was determined as follows: a 3:1 (*w*/*w*) frass-to-straw ratio, supplemented with 3.08% potato starch and 1.05% yeast powder, with a 15.03% inoculum size, 65.05% moisture content, and an initial pH of 7.0, fermented at 30 °C for 6 days. Under these conditions, the myxospore concentration reached 6.61 × 10^7^ CFU/g, representing a 131.2-fold increase compared to unoptimized conditions (5.0 × 10^5^ CFU/g). Greenhouse pot trials showed that the optimized KS01 solid agent achieved a control efficacy of 71.9%. In field trials conducted in heavily infested soil, the agent maintained control efficacies of 71.2% at the budding stage and 54.5% at the bolling stage, significantly outperforming the commercial fungicide Benziothiazolinone (51.4% and 41.4%, respectively) and the sterile substrate control. Furthermore, application of the KS01 agent significantly promoted cotton growth, with seed cotton yield reaching 5380.0 kg/ha, equating to a 50.4% reduction in yield loss compared to the untreated control. Our results demonstrate that the valorization of *P. brevitarsis* frass through optimized SSF significantly enhances the production and field performance of *M. fulvus* KS01. This study provides a novel technical framework and a robust microbial resource for the sustainable management of Verticillium wilt in saline alkali cotton production systems.

## 1. Introduction

Cotton Verticillium wilt, caused by the soil-borne fungus *Verticillium dahliae* Kleb., is one of the most devastating diseases in global cotton production. Due to its high pathogenicity, rapid dispersal, and extreme persistence, it is frequently termed the “cancer of cotton” [1,2]. The pathogen survives in the soil for extended periods as microsclerotia, which are highly resistant to traditional crop rotation and chemical fumigation. Furthermore, resistance breeding is often hampered by the rapid evolution of pathogenic races [3]. The long-term, intensive use of synthetic fungicides has not only exacerbated pathogen resistance but also posed significant threats to agro-ecosystems and human health [4]. Consequently, the development of sustainable, efficient, and environmentally friendly biological control agents (BCAs) has become a priority for the integrated management of cotton Verticillium wilt.

Myxobacteria, a group of high-order prokaryotes, have gained significant attention for their unique “social” behaviors and biocontrol potential [5]. Myxobacteria employ a “wolf-pack” hunting strategy to actively prey on soil bacteria and fungi while secreting a diverse repertoire of secondary metabolites, including antibiotics and hydrolytic enzymes, to suppress pathogen growth [6,7]. Occupying the apex of the soil microbial food web, their predatory activity directly modulates the soil micro-environment, playing a pivotal role in maintaining ecological balance and promoting plant health [8]. These characteristics provide myxobacteria with unique competitive advantages as a novel class of BCAs. They have demonstrated promising results in managing various plant diseases, such as fire blight, pepper anthracnose, tomato bacterial wilt, and cucumber Fusarium wilt [9,10,11,12]. Crucially, under nutrient-depleted or stressful conditions, myxobacteria differentiate into highly resilient myxospores [13]. Myxospores are resistant to desiccation, high temperatures, and UV radiation, enabling long-term survival in soil [14]. This trait makes myxobacteria particularly suitable for the development of solid microbial formulations, overcoming the shelf-life and stability limitations common in liquid fermentations.

The effective control of soil-borne pathogens typically requires the direct application of agents into the soil. BCAs are primarily produced through either submerged (liquid) fermentation or solid-state fermentation (SSF). Liquid fermentation is often limited by high equipment investment, complex downstream processing, and short shelf-life [15]. In contrast, SSF offers advantages such as lower infrastructure and material costs, simplified processing, environmental sustainability, and ease of storage and transport [16]. Regarding substrate selection, utilizing agricultural waste as a substitute for expensive raw materials can significantly reduce production costs [17]. Insect frass—the byproduct of organic waste conversion by insects is rich in organic matter, available nitrogen, phosphorus, potassium, and chitin. It is increasingly recognized as a novel bio-organic fertilizer with both nutritional and disease-suppressive potential [18].

Previously, our research group isolated and screened *Myxococcus fulvus* KS01 from saline alkali cotton fields in Xinjiang, which exhibited potent inhibitory activity against *V. dahliae*. In greenhouse pot trials, root drenching with liquid culture achieved a control efficacy of 35.05%. While a preliminary solid agent developed using *P. brevitarsis* frass as a substrate showed improved performance, the myxospore concentration reached only 10^5^ CFU/g, limiting its shelf-life and field efficacy [19]. Given that high myxospore concentrations are critical for consistent biocontrol efficacy, this study proposes the use of insect frass as a primary substrate for the solid-state fermentation of myxobacteria to maximize myxospore yield. We optimized the SSF process for strain KS01 and systematically evaluated the biocontrol efficacy of the optimized solid agent against cotton Verticillium wilt, as well as its impact on cotton yield, through both greenhouse pot experiments and field trials.

## 2. Materials and Methods

### 2.1. Strains, Media, and Plant Materials

*V*. *dahliae* (Vd, KC282468) was kindly provided by Dr. Aixing Gu of Xinjiang Agricultural University. *Myxococcus fulvus* KS01 was isolated from saline alkali cotton fields in Yuli County, Xinjiang, China [19].

The activation of *M. fulvus* was performed on VY/2 agar, and liquid cultures were maintained in LBS medium. The pathogenic fungus was cultured for sporulation on Potato Dextrose Agar (PDA) and Czapek Dox medium. The compositions of LBS (7 g/L soluble starch, 5 g/L yeast extract, 1 g/L casitone, 1 g/L MgSO4·7H_2_O, pH 7.0), VY/2 (5 g/L yeast, 1 g/L CaCl_2_·2H_2_O, 15 g/L agar, pH7.2), PDA, and Czapek Dox media were prepared as described by Han et al. [20].

The cotton variety used was Gossypium hirsutum cv. “X19075”, a non-transgenic, early maturing variety resistant to Fusarium wilt and tolerant to Verticillium wilt [21]. Seeds were provided by Qiang Sheng from the Bayingolin Agricultural Research Institute, Xinjiang.

### 2.2. Screening of Solid-State Fermentation (SSF) Substrates

Strain KS01 was activated on VY/2 plates and transferred to LBS broth for 2 d (30 °C, 180 rpm). Subsequently, a 7% (*v*/*v*) inoculum was added to fresh LBS medium (125 mL/250 mL flask) and cultured for 72 h at 30 °C and 180 rpm. The cell density was adjusted to an OD_600_ of 2.0 to serve as the seed inoculum for SSF. Four substrates were evaluated: *P*. *brevitarsis* frass, yellow mealworm (*Tenebrio molitor*) frass, rabbit manure, and sheep manure. Each was mixed with wheat straw (length = 1 cm) at a mass ratio of 3:1 to form the base medium. Following autoclaving, the pH was adjusted to 7.0. The seed inoculum was added at 5% (*v*/*w*) and the moisture content was adjusted to 60%. Fermentation was conducted at 30 °C for 8 d. Starting from day 4, 1.0 g samples were collected daily, suspended in 9 mL sterile water, and subjected to ultrasonic treatment (950 W, 30% output, 3 s pulse/10 s interval for 5 min), incubated 2 h in 50 °C water bath. Myxospore concentration (CFU/g) was determined via serial dilution and plating on VY/2 agar [20]. Each treatment was performed in triplicate.

### 2.3. Single-Factor Optimization of Media Components

Aiming to enhance myxospore productivity, we supplemented the frass-based medium with wheat straw and various carbon and nitrogen adjuvants. Using the selected *P. brevitarsis* frass and wheat straw mixture (3:1 *w*/*w*) as the base, various carbon sources (potato starch, sucrose, and dextrin) were added at concentrations of 0, 0.5, 1, 3, and 5% (*w*/*w*). After sterilization, conditions were set to 60% moisture, 5% (*v*/*w*) inoculum, 30 °C, and initial pH 7.0. Myxospore counts were measured after 6 d. Subsequently, the optimal carbon source was fixed, and nitrogen sources (yeast powder, soybean peptone, and beef extract) were evaluated at the same concentrations (0–5% *w*/*w*) in triplicate.

### 2.4. Optimization of Culture Conditions

Based on the optimized carbon and nitrogen sources, the fermentation conditions were refined using a one-factor-at-a-time (OFAT) approach. The baseline conditions were 60% moisture, 5% (*v*/*w*) inoculum, 30 °C, initial pH 7.0, and a 6-day incubation period. The variables tested included: initial pH (6.7, 7.0, 7.3, 7.6, 7.9), temperature (27, 30, 34, 38 °C), moisture content (55, 60, 65, 70, 75%), and inoculum size (1, 5, 10, 15, 20%). The pH of the solid-state fermentation (SSF) medium was adjusted prior to sterilization. Briefly, a representative sample of the solid substrate was mixed with sterile distilled water at a ratio of 1:10 (*w*/*v*). The mixture was stirred thoroughly and allowed to equilibrate for 30 min before the pH of the supernatant was measured using a pH meter. The target pH was achieved by incrementally amending the solid substrate with either 1 mol/L NaOH or 1 mol/L HCl. Final pH was verified post-sterilization to ensure stability. Each treatment was performed in triplicate, with myxospore count as the evaluation index.

### 2.5. Response Surface Optimization Experiments

#### 2.5.1. Plackett Burman (PB) Tests

Based on the results of the single-factor optimization experiments, further optimization was conducted through Plackett Burman (PB) tests. Design-Expert 13.0 software was used to design the experiments [22]. Each factor was set at two levels within the interval, namely the high level “+1” and the low level “−1”, and each group of experiments was repeated three times. Taking the number of myxospores in the solid microbial agent after fermentation as the response value, the main factors affecting the growth of strain KS01 were screened from the carbon source, nitrogen source, initial pH, incubation temperature, moisture content, and seed inoculum volume.

#### 2.5.2. Box Behnken Design (BBD) Tests

Utilizing the principles of the Box Behnken Design (BBD) experimental design [23], the number of myxospores was taken as the response value, with the determination method being the same as described in Section 2.2. Taking the optimal parameters obtained from the single-factor experimental optimization as the center point, the Design-Expert 13.0 software was used to design a 4-factor, 3-level experiment consisting of 29 groups [24], and to analyze and process the results.

#### 2.5.3. Verification Experiments for Response Surface Optimization Results

The method described in Section 2.2 was used to determine the number of myxospores in the solid microbial agent under the optimized fermentation conditions. The number of myxospores obtained under the initial fermentation conditions (base fermentation medium of *P. brevitarsis* frass and wheat straw mixed uniformly at a mass ratio of 3:1, moisture content 60%, 5% seed inoculum volume, incubation temperature 30 °C, initial pH 7.0, and culture for 6 d) was used as the control.

### 2.6. Greenhouse Biocontrol Efficacy Trials

*V. dahliae* was cultured on PDA for 7 d at 28 °C. Four mycelial plugs (d = 5 mm) were inoculated into 200 mL Czapek Dox broth and shaken at 200 rpm for 6 d at 28 °C. Spores were collected via filtration through four layers of sterile gauze and adjusted to 1.0 × 10^8^ conidia/mL [20].

Cotton seedlings were prepared following the reference [25]. Five treatments were established: (1) USFK + Vd: 1.0 g unoptimized *M. fulvus* KS01 solid agent applied under each seed. (2) OSFK + Vd: 1.0 g optimized *M. fulvus* KS01 solid agent applied under each seed. (3) SSFS + Vd: 1.0 g sterile solid fermentation substrate applied under each seed. (4) Chemical Control (CC) + Vd: No substrate at sowing; root-drenched with 5 mL of 1.5% Benziothiazolinone (800× dilution) (Shaanxi Xida Huaite Technology Industrial Co., Ltd., Xi’an, China) at the two-leaf stage. (5) Negative Control (Vd): Inoculated with *V. dahliae* only.

All groups were inoculated at the three-leaf stage by root wounding and application of 5 mL/plant of the *V. dahliae* spore suspension. Each treatment included three replicates (10 plants per pot). Disease severity was recorded 35 days post-inoculation (dpi) using a five-grade scale [26]. Disease Index (DI) and Control Efficacy (CE) were calculated as follows:

DI = [∑(No. of diseased plants in each grade × Grade value)/(Total plants × Highest grade value)] × 100(1)

CE (%) = (DI of Control − DI of Treatment)/DI of Control × 100(2)

### 2.7. Field Biocontrol Efficacy Trials

Field trials were conducted in a *Verticillium*-infested nursery in Bayingolin Agricultural Research Institute, Xinjiang (86°07′24.53″ E, 41°44′24.97″ N). The experimental site was characterized by typical saline alkali soil with the following physico-chemical properties: pH 8.62, electrical conductivity (EC) of 208.63 μs/cm, organic matter content of 23.64 g/kg, available phosphorus (P) of 99.60 mg/kg, available potassium (K) of 213.00 mg/kg, and total nitrogen (TN) of 1.08 g/kg. Plots were 8 m × 1.5 m with four rows (wide-narrow spacing: 66 cm and 10 cm; plant spacing: 10 cm). Treatments included: (1) SFK (solid fermentation of KS01): 21 g/m optimized KS01 solid agent applied in the seed furrow; (2) SSFS (sterile solid fermentation substrate): 21 g/m sterile substrate; (3) Chemical Control: 40 mL/plant of 1.5% Benziothiazolinone (800×) at the three-leaf stage, repeated after 14 d; (4) CK: Untreated control. A randomized block design with four replicates was used. Conventional management was applied without additional fertilizers or fungicides. Disease surveys were conducted during the budding and bolling stages [27]. Agronomic traits and seed cotton yield were recorded at harvest. The yield enhancement compared to natural infested control was calculated as

Yield Enhancement (%) = (Treatment Yield − CK Yield)/CK Yield × 100(3)

### 2.8. Statistical Analysis

Data were analyzed using SPSS Statistics 22.0 (IBM, Armonk, NY, USA). Means, standard deviations (SD), and relative standard deviations (RSD) were determined. Design-Expert 13.0 was used for RSM design and analysis. Statistical significance was assessed using paired *t*-tests with Bonferroni’s correction, with *p* < 0.05 considered significant. Figures were generated using GraphPad Prism 8.0.2 [28]. Data from single-factor experiments and pot trials were analyzed using one-way analysis of variance (ANOVA). Significant differences between treatments were determined using Duncan’s multiple range test (*p* < 0.05), with different lowercase letters indicating statistical significance. For the response surface methodology (RSM) experiments, ANOVA was employed to evaluate the significance of the model and individual factors based on *F*-statistics and *p*-values.

## 3. Results

### 3.1. Screening of Initial Fermentation Substrates

**Figure 1:**
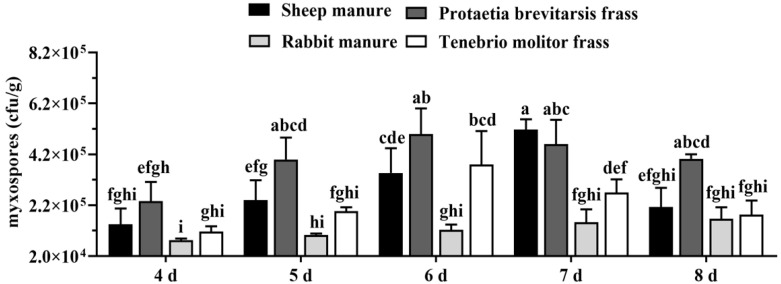
Effects of different solid fermentation substrates on myxospore yield. Data are means ± SD of three replicates. Means followed by different letters are significantly different (Duncan’s multiple range test, *p* < 0.05).

Under identical fermentation conditions, the colonization and growth of strain KS01 were evaluated using four different base substrates, sheep manure, rabbit manure, *P. brevitarsis* frass, and yellow mealworm (*Tenebrio molitor*) frass, each mixed with wheat straw. As shown in Figure 1, strain KS01 successfully colonized all four substrates. In both *P. brevitarsis* and yellow mealworm frass media, myxospore yields peaked on day 6 at 5.00 × 10^5^ CFU/g and 3.80 × 10^5^ CFU/g, respectively. While sheep manure reached a maximum of 5.17 × 10^5^ CFU/g on day 7, the concentration plummeted to 2.13 × 10^5^ CFU/g by day 8. Rabbit manure yielded the lowest peak of 1.67 × 10^5^ CFU/g on day 8. Considering fermentation efficiency and the natural granular texture of *P. brevitarsis* frass—which is conducive to mechanical application—it was selected as the optimal base substrate with a 6-day fermentation period.

### 3.2. Screening of Supplemental Carbon and Nitrogen Sources

**Figure 2:**
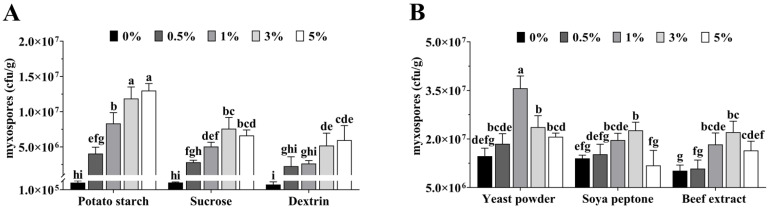
Effects of different concentrations of carbon and nitrogen sources on myxospore yield in solid microbial agents. (**A**) Different carbon sources. (**B**) Different nitrogen sources. Data are means ± SD of three replicates. Means followed by different letters are significantly different (Duncan’s multiple range test, *p* < 0.05).

The addition of various carbon sources to the *P. brevitarsis* frass straw base significantly enhanced myxospore production (Figure 2A). Potato starch yielded the highest results; at concentrations of 3% and 5%, myxospore counts reached 1.19 × 10^7^ CFU/g and 1.30 × 10^7^ CFU/g, respectively, with no significant difference between the two (*p* > 0.05). These yields were significantly higher than those achieved with other concentrations or with sucrose and dextrin (*p* < 0.05). Consequently, 3% potato starch was determined as the optimal carbon source.

Regarding nitrogen sources (Figure 2B), the addition of 1% yeast powder resulted in a myxospore count of 3.57 × 10^7^ CFU/g, which was significantly superior to all other concentrations and sources tested (soybean peptone and beef extract, *p* < 0.05). Thus, 1% yeast powder was selected as the optimal nitrogen source.

### 3.3. Single-Factor Optimization of Fermentation Conditions

**Figure 3:**
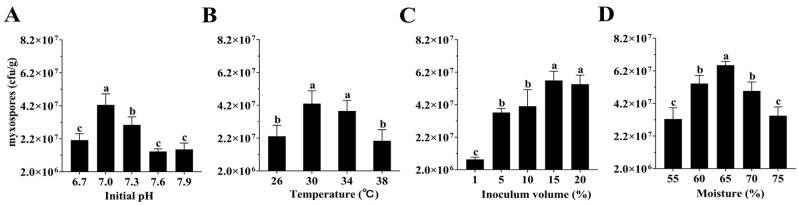
Effects of different environmental fermentation conditions on myxospore yield in solid microbial agents. (**A**) Initial pH. (**B**) Incubation temperature. (**C**) Inoculum volume. (**D**) Medium moisture content. Data are means ± SD of three replicates. Means followed by different letters are significantly different (Duncan’s multiple range test, *p* < 0.05).

Using the optimized base medium (frass/straw + 3% potato starch + 1% yeast powder), fermentation conditions were further refined. Initial pH: Myxospore yield peaked at 4.25 × 10^7^ CFU/g at pH 7.0. Deviations below or above this value led to significant declines in production (Figure 3A). Temperature: Strain KS01 grew between 26 °C and 38 °C, with a maximum yield of 4.30 × 10^7^ CFU/g at 30 °C. Yields decreased significantly at temperatures above 34 °C (Figure 3B). Inoculum Size: Spore yields increased with inoculum size up to 15%, reaching 5.69 × 10^7^ CFU/g. No significant further increase was observed at 20% (Figure 3C). Moisture Content: Yields increased with moisture levels between 55% and 65%, peaking at 6.54 × 10^7^ CFU/g at 65% moisture. Moisture levels exceeding 65% resulted in a significant reduction in yields (Figure 3D).

The resulting optimal parameters were initial pH 7.0, 30 °C, 15% inoculum size, and 65% moisture content.

### 3.4. Response Surface Optimization for Myxospore Production

#### 3.4.1. *Optimized Nutrient and Cultural Conditions*

Six factors (potato starch, yeast powder, initial pH, temperature, inoculum size, and moisture content) were evaluated. Myxospore counts across 12 runs ranged from 0.59 × 10^7^ to 3.86 × 10^7^ CFU/g (Supplementary Table S1). The model showed high reliability (R^2^ = 94.87%, adjusted R^2^ = 88.71%, *p* = 0.0044). All factors had a positive impact, with potato starch, yeast powder, inoculum size, and moisture being the most significant (*p* < 0.05). Based on *F*-values, the order of influence was potato starch > yeast powder > moisture content > inoculum size > initial pH > temperature (Table 1). These top four variables were selected for the Box Behnken Design (BBD).

#### 3.4.2. *Optimized Combination Parameters*

BBD was performed using the optimized points from the single-factor tests as center points (2.5–3.5% potato starch, 0.5–1.5% yeast powder, 12–18% inoculum, and 62–68% moisture) (Supplementary Table S2). The ANOVA for the regression model (Table 2) indicated the model was highly significant (*p* < 0.0001, R^2^ = 98.67%). The Lack of Fit was non-significant (*p* = 0.5403), confirming model predictive accuracy. Quadratic terms (A^2^, B^2^, C^2^, D^2^) and interactions AB and BC had highly significant effects (*p* < 0.01), while BD and CD were significant (*p* < 0.05). The final quadratic equation was

Y = 6.33 + 0.5492A + 0.1958B − 0.0575C + 0.0458D + 0.9675AB + 0.2675AC + 0.0025AD + 0.5050BC + 0.3700BD + 0.3850CD − 2.14A^2^ − 1.79B^2^ − 2.00C^2^ − 2.54D^2^ (4)

**Figure 4:**
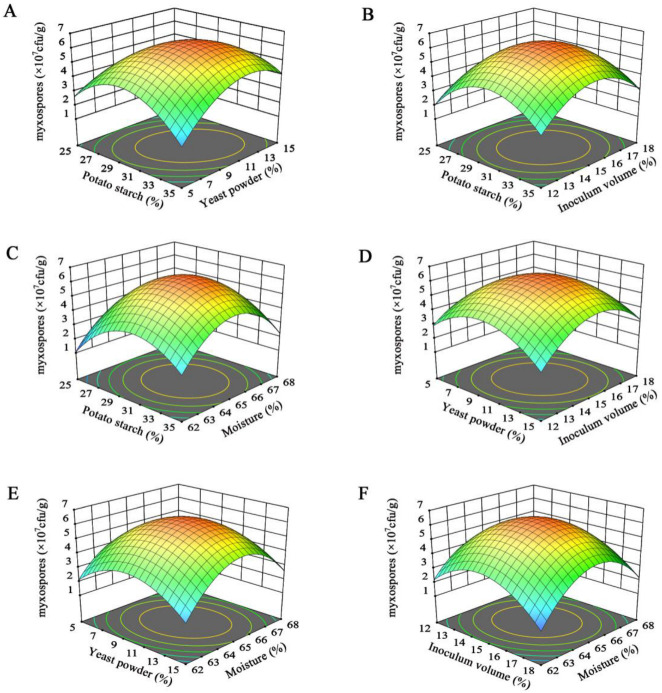
Response surface optimization of the effects of media carbon/nitrogen sources and fermentation conditions on myxospore yield. (**A**) Potato starch × Yeast powder. (**B**) Potato starch × Inoculum volume. (**C**) Potato starch × Moisture content. (**D**) Yeast powder × Inoculum volume. (**E**) Yeast powder × Moisture content. (**F**) Inoculum volume × Moisture content.

We used 3D response surface plots (Figure 4) to visualize these interactions. The surfaces for potato starch × yeast powder (AB) and yeast powder × inoculum size (BC) were notably steep (Figure 4A,D), indicating strong synergistic interactions. In contrast, the interactions between potato starch and seed inoculum volume, potato starch and medium moisture content, yeast powder and medium moisture content, and seed inoculum volume and medium moisture content were relatively weak (Figure 4B,C,E,F).

#### 3.4.3. Validation of Optimized Conditions

Design-Expert software was used for data analysis and solving the regression equation. After conversion, the optimal levels for the four factors were determined as follows: potato starch 3.08%, yeast powder 1.05%, seed inoculum volume 15.03%, and medium moisture content 65.05%. Under these conditions, the model predicted a maximum response value (Y) of 6.38 × 10^7^ CFU/g for the number of myxospores in the solid microbial agent. Three replicate verification experiments were conducted according to the optimal fermentation conditions predicted by the model. The actual obtained myxospore count was 6.61 × 10^7^ CFU/g, with a relative error of 3.48% compared to the predicted value, confirming the reliability of the model. Furthermore, compared to the myxospore count of 5.00 × 10^5^ CFU/g in the solid microbial agent before optimization (Section 3.1), the yield increased by 131.2 times, demonstrating significant practical value.

Through single-factor optimization experiments and response surface optimization, the optimal fermentation conditions for strain KS01 were determined: using a mixture of *P*. *brevitarsis* frass and wheat straw (3:1 *w*/*w*) as the base fermentation medium, with the addition of 3.08% potato starch and 1.05% yeast powder; after thorough sterilization, the culture was maintained for 6 d at 30 °C with a seed inoculum volume of 15.03%, medium moisture content of 65.05%, and an initial pH of 7.0.

### 3.5. Greenhouse Biocontrol Efficacy of Optimized KS01 Solid Agent

Dissection of cotton stems revealed that KS01 treatment significantly reduced the incidence and Disease Index (DI) of Verticillium wilt (Table 3). The unoptimized KS01 agent achieved 63.8% control efficacy, whereas the optimized agent reached 71.9%. This performance was significantly superior to the chemical control (Benziothiazolinone, 54.8%). Interestingly, the sterile substrate (SSFS) also provided some protection (33.3% efficacy).

### 3.6. Field Biocontrol Efficacy

Field trials confirmed severe disease pressure in the control group (DI of 29.5 at budding and 44.3 at bolling stage). Application of the optimized KS01 agent significantly mitigated the disease, with control efficacies of 71.2% (budding stage) and 54.5% (bolling stage) (Table 4). These results were significantly better than Benziothiazolinone (51.4% and 41.4%) and the sterile substrate (44.6% and 35.0%).

### 3.7. Impact on Cotton Agronomic Traits and Seed Cotton Yield

While *Verticillium* wilt significantly inhibited growth in the control group, KS01 treatment significantly improved plant height, stem diameter, boll number, and fruit branch number (*p* < 0.05, Table 5). At harvest, the control yield (natural infected) was 3577.5 kg/ha, while the KS01 treatment achieved the highest yield of 5380.0 kg/ha—representing a 50.4% yield enhancement compared to the control. Yields for the sterile substrate and chemical control were 4307.5 kg/ha and 4117.5 kg/ha, respectively.

## 4. Discussion

Myxobacteria are characterized by a sophisticated social life cycle. Under nutrient deprivation or environmental stress, vegetative cells aggregate to form fruiting bodies and differentiate into myxospores [5]. As dormant structures, myxospores feature thick cell walls and low metabolic activity, granting them exceptional resistance to desiccation, high temperatures, UV radiation, and chemical agents [14,29]. Studies have demonstrated that while vegetative cells perish quickly once removed from a growth medium, myxospores can remain viable for years or even decades [30]. From a practical application standpoint, this high resilience ensures that solid-state fermentation (SSF) agents maintain high viable counts during storage, transport, and application, thereby extending product shelf-life. Furthermore, a high concentration of myxospores enhances the competitive colonization of myxobacteria in the soil, improving survival rates and long-term persistence within complex microbial ecosystems [31]. Our greenhouse trials confirmed this: the optimized agent (10^7^ CFU/g) achieved a control efficacy of 71.9%, significantly higher than the unoptimized agent (10^5^ CFU/g, 63.8%), underscoring that maximizing myxospore density is critical for robust biocontrol.

The selection of an appropriate SSF substrate is a decisive factor in both agent quality and production cost. In this study, we evaluated sheep manure, rabbit manure, and two types of insect frass. While *M. fulvus* KS01 colonized all substrates, the *P*. *brevitarsis* frass-based medium yielded peak myxospore counts by day 6. Beyond nutrition, this substrate offers a natural granular structure that facilitates moisture regulation and mechanical field application. Previous research noted that solid agents of *Corallococcus* sp. EGB using rabbit manure outperformed liquid cultures against cucumber Fusarium wilt [12]. Similarly, combining myxobacteria with organic fertilizers has shown superior efficacy over pure cultures in tobacco-rice rotations [32]. Our findings align with these observations: the KS01 solid agent significantly outperformed liquid root drenching. This suggests that insect frass provides an ideal nutritional and physical matrix that mimics the natural niche of myxobacteria, promoting both colonization and sporulation. Additionally, the gradual decomposition of the organic matrix in soil likely provides a sustained nutrient supply, extending the “bio-barrier” effect against pathogens.

Nutritional inputs such as carbon and nitrogen sources are fundamental regulators of microbial development [33]. We found that 3% potato starch and 1% yeast powder were optimal for myxospore production. Sporulation in myxobacteria is a tightly regulated developmental program typically triggered by nutrient exhaustion [34,35]. Compared to sucrose or dextrin, the slow-release nature of potato starch likely creates a “progressive nutrient limitation” environment that supports initial biomass accumulation while effectively inducing high-efficiency sporulation [36]. Yeast powder, rich in amino acids, vitamins, and growth factors, serves as a high-quality organic nitrogen source. Since social behaviors and sporulation require substantial nitrogen, the rapid assimilation of yeast powder satisfies the high-density growth requirements of strain KS01. While the addition of carbon and nitrogen sources increased the initial raw material costs of solid-state fermentation, the 131-fold increase in productivity significantly reduced the unit cost per myxospore, demonstrating superior cost-effectiveness for large-scale production. As higher prokaryotes, the growth and fruiting body formation of myxobacteria are highly sensitive to environmental factors [14,37,38]. Our results indicate that pH 7.0 is optimal, consistent with the preference of most soil myxobacteria for neutral or slightly alkaline conditions [39,40]. Extreme pH levels likely inhibit membrane-associated enzymes, hindering cell aggregation and differentiation. Furthermore, the optimal moisture content of 65% balances nutrient solubility with gas exchange; excessive moisture leads to substrate compaction and hypoxia, while insufficient moisture limits nutrient diffusion [41]. Notably, a high inoculum size (15%) was found to be beneficial. This is likely due to the “social” nature of myxobacteria—their gliding motility and “wolf-pack” hunting strategy are density-dependent behaviors regulated by quorum sensing [42]. A higher initial density may shorten the lag phase, allowing the population to reach the quorum-sensing threshold faster and accelerating the transition to sporulation [43]. However, increasing the inoculum to 20% offered no further benefit, likely due to rapid nutrient depletion and limited aeration in the dense culture.

While conventional biocontrol agents such as *Trichoderma* spp. and *Bacillus* spp. are widely utilized, myxobacteria offer distinct physiological mechanisms for plant disease suppression. In this study, strain KS01 was identified from a library of 105 isolates as a superior candidate, surpassing other myxobacteria through its exceptional myxospore productivity and robust efficacy against *Verticillium* wilt in both in vitro plate assays and in vivo greenhouse trials [19]. In field trials conducted in heavily infested cotton fields, the KS01 solid agent outperformed the chemical fungicide Benziothiazolinone (51.4%) and SSFS (sterile solid fermentation substrate) (33.3%). While insect frass provides a supportive foundation for pathogen suppression, the high productivity of strain KS01 is the critical factor in the formulation’s success, suggesting a potent synergy between the substrate and the myxobacterium in field applications. While the control efficacy was 71.2% during the budding stage, it declined to 54.5% by the bolling stage. This decline likely reflects the increasing infection pressure of *V. dahliae* as the season progresses and the challenges of maintaining high population densities in a complex field environment influenced by fluctuating temperature, moisture, and native microbial competition [44]. Future strategies to enhance field persistence could include multiple applications or integration with other sustainable management practices.

The SSF process established here for *M. fulvus* KS01 is characterized by simple components and stable efficacy, making it suitable for industrial scale-up. Nevertheless, the transition to large-scale production will depend on market demand; furthermore, the practical operational protocols and overall cost-efficiency must be validated through pilot-scale trials in an industrial setting. By achieving myxospore concentrations exceeding 10^7^ CFU/g, this agent provides a potent new tool for the green management of Verticillium wilt in saline alkali cotton regions.

## 5. Conclusions

This study successfully optimized a solid-state fermentation process for *Myxococcus fulvus* KS01 using a mixture of *P*. *brevitarsis* frass and wheat straw (3:1 *w*/*w*) as a base substrate. Through single factor experiments and Response Surface Methodology, we determined the optimal conditions to be 3.08% potato starch, 1.05% yeast powder, 15.03% inoculum size, 65.05% moisture, and an initial pH of 7.0. Under these parameters, the myxospore concentration reached 6.61 × 10^7^ CFU/g, representing a 131.2-fold increase over unoptimized conditions. The resulting solid agent demonstrated robust biocontrol efficacy against cotton Verticillium wilt in both greenhouse (71.9%) and field (up to 71.2%) trials, while significantly reducing yield loss by 50.4%. These results highlight the potential of insect frass-based myxobacterial formulations as a sustainable and effective biological alternative to synthetic fungicides in cotton production, particularly in saline alkali field conditions.

## Figures and Tables

**Table 1:** Analysis of variance (ANOVA) for the Plackett Burman (PB) test results.

Source	Coefficient	Stdized Effect	Contribution/%	Sum of Squares	Mean Square	*F*	*p*	Importance Ranking
A	0.5592	1.118	46.20	3.75	3.75	44.82	0.0011	1
B	0.3958	0.792	23.15	1.88	1.88	22.46	0.0052	2
C	0.0608	0.122	0.55	0.0444	0.0444	0.5305	0.4991	6
D	0.1492	0.298	3.29	0.2670	0.2670	3.19	0.1342	5
E	0.2642	0.528	10.31	0.8374	0.8374	10.00	0.0250	4
F	0.2825	0.565	11.79	0.9577	0.9577	11.44	0.0196	3
Model	2.46	0	0	7.74	1.29	15.41	0.0044	

Note: A (Potato starch); B (Yeast powder); C (Temperature); D (pH); E (Inoculation amount); F (Moisture).

**Table 2:** Analysis of variance (ANOVA) for the Box Behnken Design (BBD) test results.

Source	Sum of	df	Mean	*F*	*p*	Distinctiveness
Model	86.69	14	6.19	74.37	<0.0001	**
A	3.62	1	3.62	43.43	<0.0001	**
B	0.4602	1	0.4602	5.52	0.0340	*
C	0.0397	1	0.0397	0.4761	0.5015	
D	0.0252	1	0.0252	0.325	0.591	
AB	3.74	1	3.74	44.93	<0.0001	**
AC	0.2862	1	0.2862	3.43	0.0850	
AD	0.0000	1	0.0000	0.0003	0.9864	
BC	1.02	1	1.02	12.24	0.003.5	**
BD	0.5476	1	0.5476	6.57	0.0225	*
CD	0.5929	1	0.5929	7.11	0.0184	*
A^2^	29.65	1	29.65	3.55.86	<0.0001	**
B^2^	20.68	1	20.68	248.19	<0.0001	**
C^2^	25.83	1	25.83	31.00	<0.0001	**
D^2^	41.71	1	41.71	500.47	<0.0001	**
Residual	1.17	14	0.0833			
Lack of Fit	0.8376	1	0.0838	1.02	0.5403	
Pure Error	0.3291	4	0.0823			
Cor Total	87.86	28				

Note: A (Potato starch); B (Yeast powder); C (Inoculation amount); D (Moisture); * *p* < 0.05, ** *p* < 0.01.

**Table 3:** Greenhouse biocontrol efficacy of KS01 solid microbial agent against cotton Verticillium wilt.

Treatment	Incidence (%)	Disease Index	Efficacy (%)
USFK + Vd	26.67 ± 2.9 b	12.7 ± 0.8 cd	63.8 ± 2.2 b
OSFK + Vd	13.33 ± 5.8 c	9.8 ± 2.3 d	71.9 ± 6.4 a
SSFS + Vd	33.33 ± 5.8 ab	23.3 ± 1.4 b	33.3 ± 4.1 d
Benziothiazolinone	30.0 ± 5.0 b	15.8 ± 1.4 c	54.8 ± 4.1 c
Vd	43.3 ± 5.8 a	35.0 ± 2.5 a	-

Note: Different letters following mean ± SD indicate significant differences analyzed by the least significant difference at 5% significance level, USFK: Unoptimized solid fermentation of KS01, OSFK: Optimized solid fermentation of KS01, SSFS: sterile solid fermentation substrate; “-” represents inability to calculate prevention effect.

**Table 4:** Field biocontrol efficacy of KS01 solid microbial agent against cotton Verticillium wilt.

Treatment	Squaring Stage			Flowering and Bolling Stage		
	Incidence %	Disease Index	Control Effect %	Incidence Rate %	Disease Index	Control Effect %
SFK	32.0 ± 2.0 b	8.5 ± 0.5 c	71.2 ± 1.7 a	50.0 ± 2.6 c	20.2 ± 1.0 d	54.5 ± 2.3 a
SSFS	49.3 ± 1.2 a	16.3 ± 0.6 b	44.6 ± 2.0 c	58.7 ± 1.5 b	28.8 ± 0.3 b	35.0 ± 0.7 c
Benziothiazolinone	33.3 ± 4.2 b	14.3 ± 0.8 b	51.4 ± 2.6 b	57.3 ± 3.1 b	26.0 ± 0.5 c	41.4 ± 1.1 b
CK	54.7 ± 4.6 a	29.5 ± 2.2 a	-	70.7 ± 1.5 a	44.3 ± 1.6 a	-

Note: Different letters following mean ± SD indicate significant differences analyzed by the least significant difference at 5% significance level, CK: No processing required; “-” represents inability to calculate prevention effect. SFK: solid fermentation of KS01. SSFS: sterile solid fermentation substrate.

**Table 5:** Effects of different treatments on cotton growth and seed cotton yield.

Treatment	Plant Height (cm)	Stem Diameter (mm)	Boll Number	Fruit Branch Number	Leaf Number	Seed Cotton Yield (kg/ha)	Yield Enhancement (%)
SKF	84.7 ± 1.5 a	11.0 ± 0.2 a	12.4 ± 0.6 a	11.7 ± 0.9 a	34.2 ± 0.8 a	5380.0 ± 192.7 a	50.4
SSFS	77.9 ± 1.3 b	10.4 ± 0.3 ab	10.5 ± 0.2 b	10.0 ± 0.7 b	32.7 ± 2.5 ab	4307.5 ± 193.6 b	20.4
Benziothiazolinone	75.5 ± 2.1 bc	10.0 ± 0.5 bc	10.3 ± 0.2 b	9.6 ± 0.1 b	29.8 ± 1.5 bc	4117.5 ± 130.1 b	15.1
CK	73.4 ± 1.0 c	9.8 ± 0.2 c	9.6 ± 0.1 c	9.2 ± 0.1 b	26.5 ± 2.4 c	3577.5 ± 97.5 c	-

Note: Different letters following mean ± SD indicate significant differences analyzed by the least significant difference at 5% significance level, CK: No processing required; “-” represents inability to calculate prevention effect. SFK: solid fermentation of KS01. SSFS: sterile solid fermentation substrate.

## Data Availability

The original contributions presented in this study are included in the article and Supplementary Materials. Further inquiries can be directed to the corresponding authors.

## Supplementary Materials

The following supporting information can be downloaded at: https://www.mdpi.com/article/10.3390/microorganisms14030610/s1, Table S1: Plackett Burman (PB) experimental design and results; Table S2: Box Behnken Design (BBD) experimental design and results.
