# Supplementary material for: Enhanced Biocontrol of Cotton Verticillium Wilt Through Optimized Solid-State Fermentation of Myxococcus fulvus KS01 Using Insect Frass as a Matrix
Source: Microorganisms. 2026 Mar 9;14(3):610. doi: 10.3390/microorganisms14030610 (PMC13028799; doi:10.3390/microorganisms14030610)
Supplement: Supplementary file 1 [file microorganisms-14-00610-s001.zip › SupplementaryTabe S2 BBD design.pdf]

Supplementary Table S2. Box-Behnken Design (BBD) experimental design and results.

Treatment	Factor				myxospores ($\times 10^7$cfu/g)
	A (%)	*B* (%)	*C* (%)	*D* (%)	
1	2.5	0.5	15	65	2.72
2	3.5	0.5	15	65	1.85
3	2.5	1.5	15	65	1.24
4	3.5	1.5	15	65	4.24
5	3	1	12	62	2.37
6	3	1	18	62	1.38
7	3	1	12	68	1.66
8	3	1	18	68	2.21
9	2.5	1	15	62	1.03
10	3.5	1	15	62	2.21
11	2.5	1	15	68	1.01
12	3.5	1	15	68	2.20
13	3	0.5	12	65	2.48
14	3	1.5	12	65	2.32
15	3	0.5	18	65	1.68
16	3	1.5	18	65	3.54
17	2.5	1	12	65	2.04
18	3.5	1	12	65	2.55
19	2.5	1	18	65	1.17
20	3.5	1	18	65	2.75
21	3	0.5	15	62	2.26
22	3	1.5	15	62	1.39
23	3	0.5	15	68	1.75
24	3	1.5	15	68	2.36
25	3	1	15	65	6.05
26	3	1	15	65	6.16
27	3	1	15	65	6.80
28	3	1	15	65	6.33
29	3	1	15	65	6.30

Note: *A* (Potato starch); *B* (Yeast extract powder); *C* (Inoculation amount); *D* (Moisture)
