# Supplementary material for: Enhanced Biocontrol of Cotton Verticillium Wilt Through Optimized Solid-State Fermentation of Myxococcus fulvus KS01 Using Insect Frass as a Matrix
Source: Microorganisms. 2026 Mar 9;14(3):610. doi: 10.3390/microorganisms14030610 (PMC13028799; doi:10.3390/microorganisms14030610)
Supplement: Supplementary file 1 [file microorganisms-14-00610-s001.zip › SupplementaryTable S1 PB design.pdf]

Supplementary Table S1. Plackett-Burman (PB) experimental design and results.

Treatment	A (%)	B (%)	C (°C)	D	E (%)	F (%)	Myxospores ($\times10^7$cfu/g)
1	3.5	1.5	28	7.2	18	68	3.86
2	2.5	1.5	32	6.8	18	68	2.57
3	3.5	0.5	32	7.2	12	68	3.05
4	2.5	1.5	28	7.2	18	62	2.57
5	2.5	0.5	32	6.8	18	68	2.15
6	2.5	0.5	28	7.2	12	68	1.4
7	3.5	0.5	28	6.8	18	62	2.55
8	3.5	1.5	28	6.8	12	68	3.43
9	3.5	1.5	32	6.8	12	62	2.58
10	2.5	1.5	32	7.2	12	62	2.13
11	3.5	0.5	32	7.2	18	62	2.65
12	2.5	0.5	28	6.8	12	62	0.59

Note: *A* (Potato starch); *B* (Yeast extract powder); *C* (Temperature); *D* (pH); *E* (Inoculation amount); *F* (Moisture)
